# Erratum: Romero-Lopez et al. Lung Metabolomics Profiling of Congenital Diaphragmatic Hernia in Fetal Rats. *Metabolites* 2021, *11*, 177

**DOI:** 10.3390/metabo11040229

**Published:** 2021-04-09

**Authors:** Maria del Mar Romero-Lopez, Marc Oria, Miki Watanabe-Chailland, Maria Florencia Varela, Lindsey Romick-Rosendale, Jose L. Peiro

**Affiliations:** 1 Center for Fetal and Placental Research, Division of Pediatric General and Thoracic Surgery, Cincinnati Chidren’s Hospital Medical Center (CCHMC), Cincinnati, OH 45229, USA; maria.romero.lopez@cchmc.org (M.d.M.R.-L.); marc.oria@cchmc.org (M.O.); maria.florencia.varela@cchmc.org (M.F.V.); 2 Perinatal Institute, Division of Neonatology, Cincinnati Children’s Hospital Medical Center, Cincinnati, OH 45229, USA; 3 Department of Surgery, College of Medicine, University of Cincinnati, Cincinnati, OH 45267, USA; 4 NMR-Based Metabolomics Core, Division of Pathology and Laboratory Medicine, Cincinnati Children’s Hospital Medical Center, Cincinnati, OH 45229, USA; Miki.Watanabe@cchmc.org (M.W.-C.); Lindsey.romick-rosendale@cchmc.org (L.R.-R.)

The authors wish to make the following corrections to this paper [1].

On page 11, in the original manuscript, Figure 6 was a repeat of Figure 5 by mistake. The following is the correct Figure 6:

## Figures and Tables

**Figure 6:**
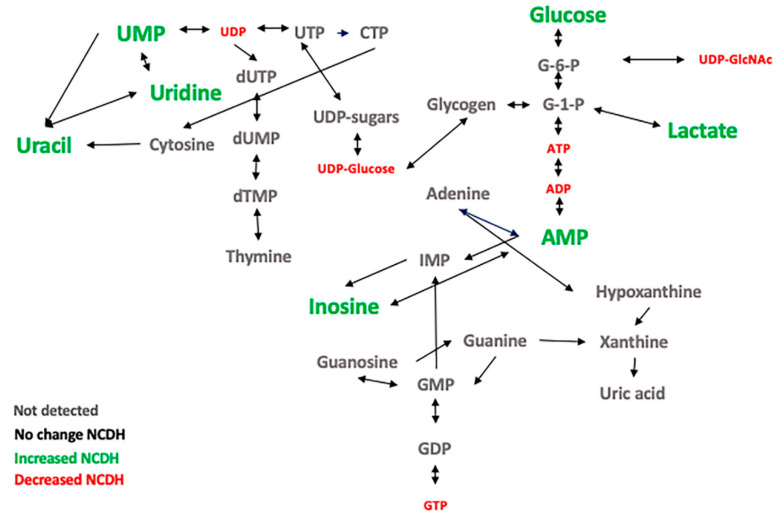
Alterations of nucleotide metabolism in fetal CDH lung. The overview of the metabolic flow of nucleotide metabolites and changes identified in the NCDH group compared with the VC group. Green (increased), red (decreased), black (no change), and grey (undetected).
