# Supplementary material for: Lung Metabolomics Profiling of Congenital Diaphragmatic Hernia in Fetal Rats
Source: Metabolites. 2021 Mar 18;11(3):177. doi: 10.3390/metabo11030177 (PMC8003001; doi:10.3390/metabo11030177)
Supplement: Supplementary file 1 [file metabolites-11-00177-s001.pdf]

Lung Metabolomics Profiling of Congenital Diaphragmatic Hernia in Fetal Rats

Maria del Mar Romero-Lopez, Marc Oria, Miki Watanabe-Chailland, Maria Florencia Varela, Lindsey Romick-Rosendale and Jose L. Peiro

Supplementary Figures

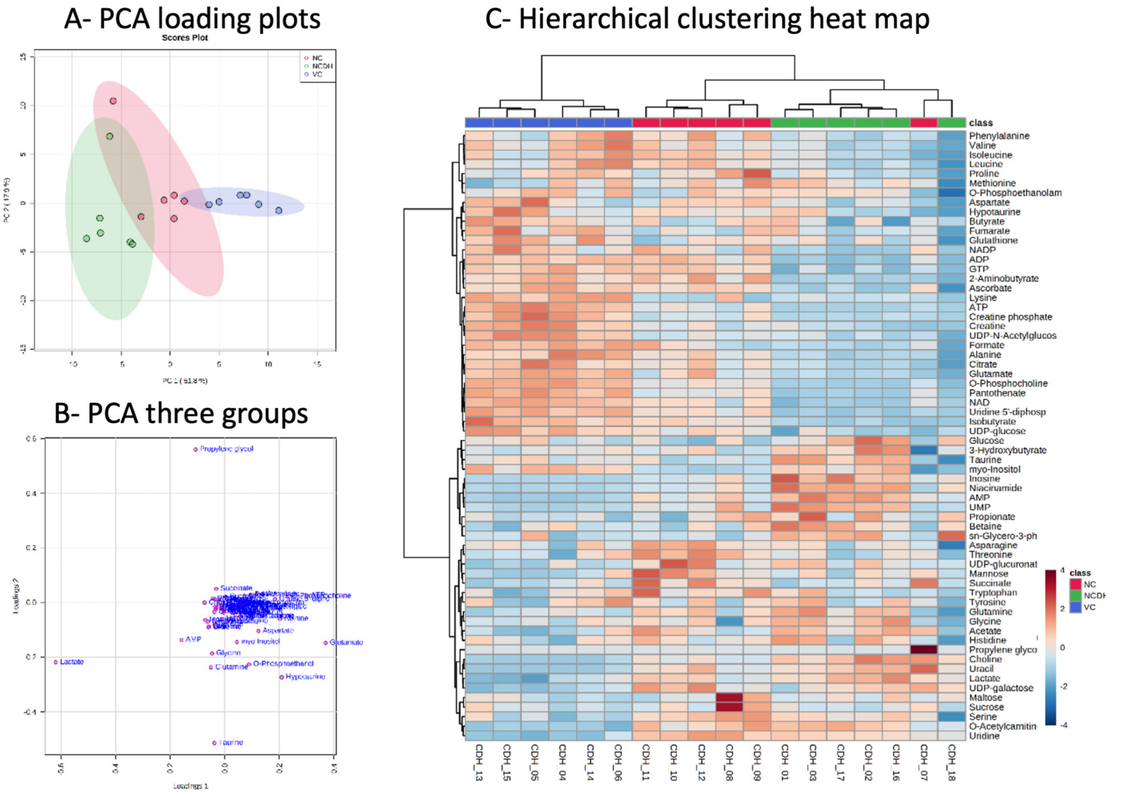

Figure S1. Assessment of CDH fetal lungs metabolic profile. A) Principal component analysis (PCA) scores plot of vehicle control (VC: blue), Nitrofen control (NC: red) and Nitrofen-CDH model (NCDH: green) with 95% confidence interval and B) the corresponding loading plots showing the unique metabolic profiles in each group. C) Hierarchical clustering heat map visualization by Euclidean distance and Ward's linkage analysis.

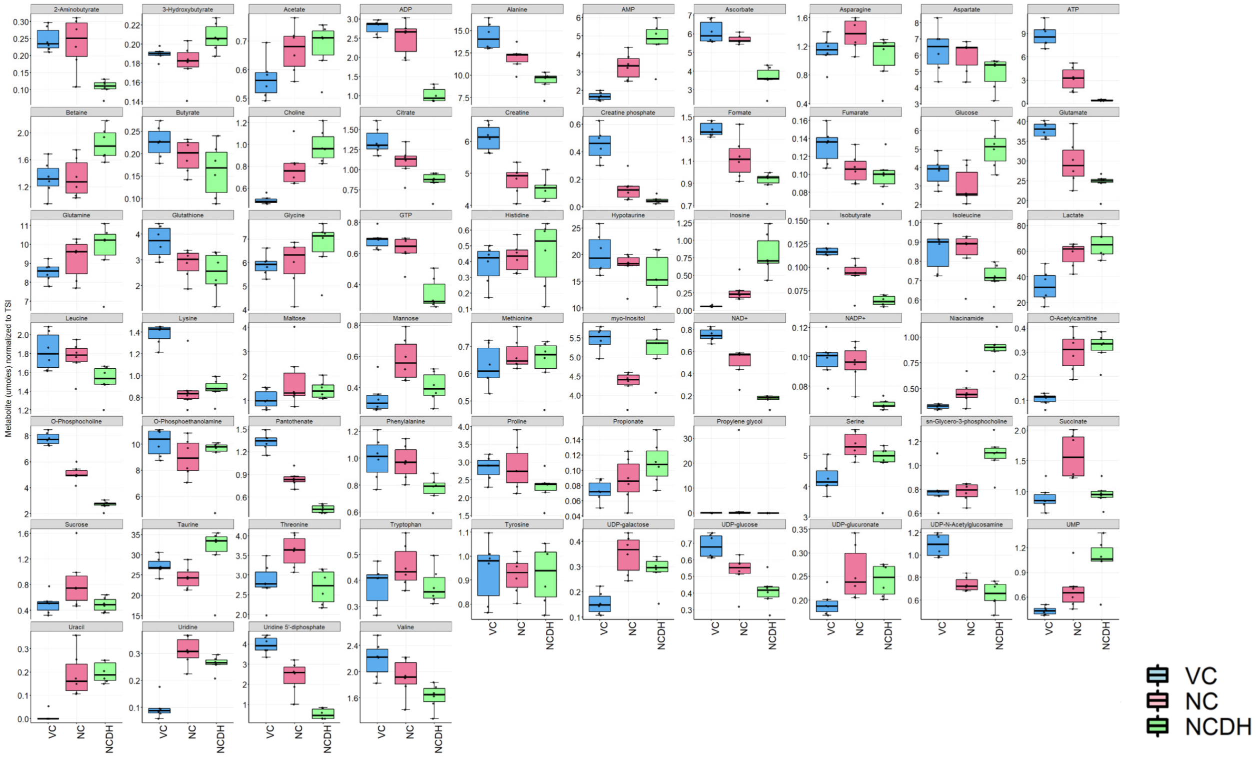

Figure S2. Concentration of metabolites normalized to total spectrum intensity (TSI) identified by H1 NMR in fetal rat lung. Box plots of 64 metabolites assessed between the treatment groups: VC-blue, NC-red, and NCDH-green.

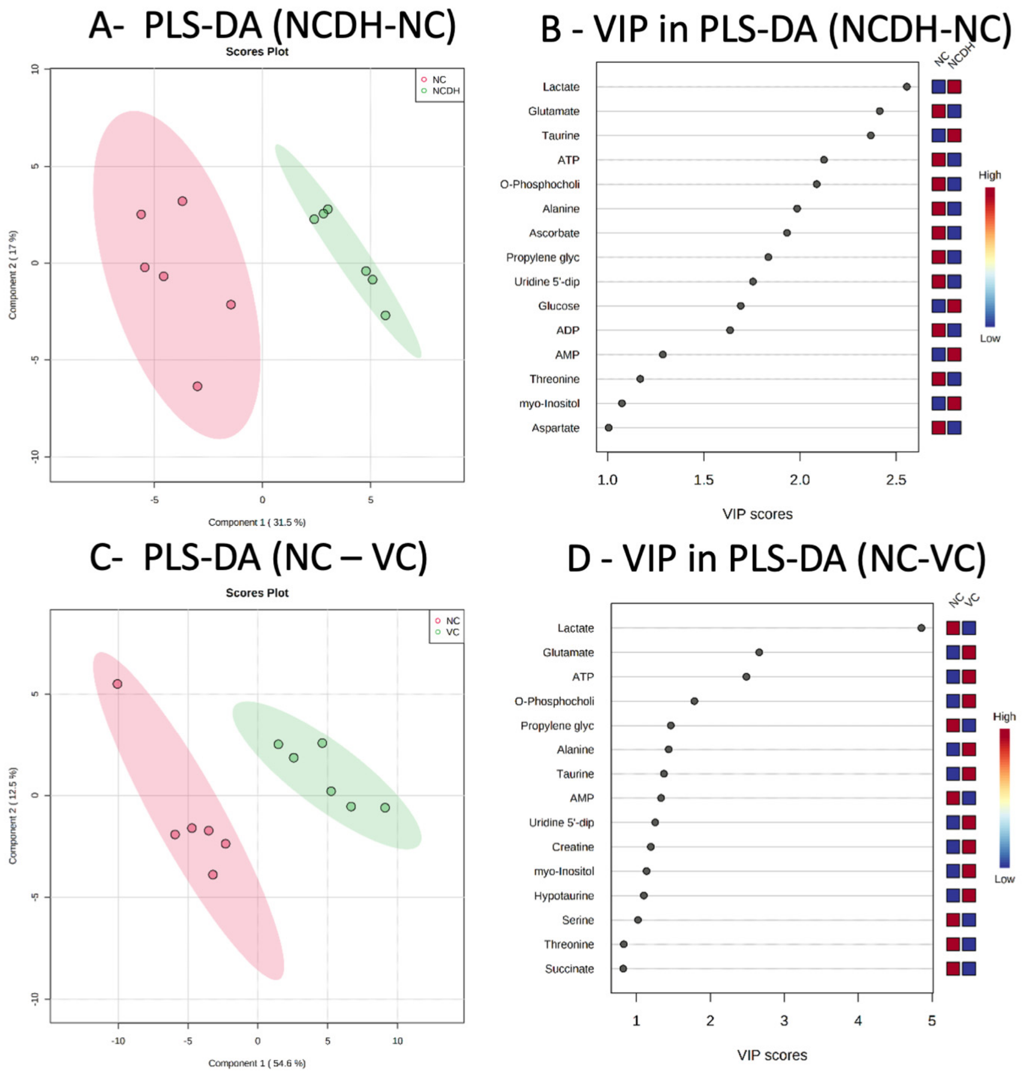

Figure S3. Comparison between NCDH-NC (effect of lung compression on the fetal lung metabolism) and NC-VC (effect of Nitrofen causing lung hypoplasia on the lung metabolism). A) Loading score plot of partial least square discriminant analysis (PLS-DA) between NCDH (green) and NC (red) groups and B) corresponding metabolites with the highest VIP-score (>2) in component 1 (EV 31.5%). C) Loading scores plot between NC (red) and VC (green) groups and D) corresponding metabolites with the highest VIP-score (>2) in component 1 (EV 54.6 %).

NCDH vs NC

Pathway Name	FDR	Impact
Starch and sucrose metabolism	0.02037	0.55373
Glycine, serine and threonine metabolism	0.031031	0.54841
Nicotinate and nicotinamide metabolism	0.03041	0.42895
Ascorbate and aldarate metabolism	4.81E-05	0.25
Pyrimidine metabolism	0.017236	0.21627
Citrate cycle (TCA cycle)	0.010713	0.15292
Inositol phosphate metabolism	0.035221	0.12939
Galactose metabolism	0.0095929	0.1085
Glycerophospholipid metabolism	0.017236	0.10756

NC vs VC

Pathway Name	FDR	Impact
Glycine, serine and threonine metabolism	7.78E-04	0.54841
Alanine, aspartate and glutamate metabolism	0.014268	0.53686
D-Glutamine and D-glutamate metabolism	0.018183	0.5
Glutathione metabolism	0.018183	0.36615
Ascorbate and aldarate metabolism	0.0026239	0.25
Histidine metabolism	0.023376	0.22131
Pyrimidine metabolism	7.78E-04	0.21627
Pentose and glucuronate interconversions	0.010071	0.20312
Glyoxylate and dicarboxylate metabolism	0.013966	0.1799
Arginine and proline metabolism	0.01433	0.17592
Cysteine and methionine metabolism	0.0087229	0.16809
Aminoacyl-tRNA biosynthesis	0.010071	0.16667
Citrate cycle (TCA cycle)	0.0061714	0.15292
Pyruvate metabolism	0.010183	0.14463
Inositol phosphate metabolism	0.0010473	0.12939
Amino sugar and nucleotide sugar metabolism	0.00102	0.12647
Arginine biosynthesis	0.021919	0.11675
Galactose metabolism	0.0030793	0.1085
Glycerophospholipid metabolism	0.0045358	0.10756

Figure S4. Functional analysis of metabolic pathways in Nitrofen-CDH model (NCDH) compared to Nitrofen control (NC) and NC compared vehicle control (VC). A) Table of metabolic pathways with the FDR corrected p-value (<0.05) from enrichment analysis and its impact factor (>0.1) from topology analysis from MetaboAnalyst4.0.

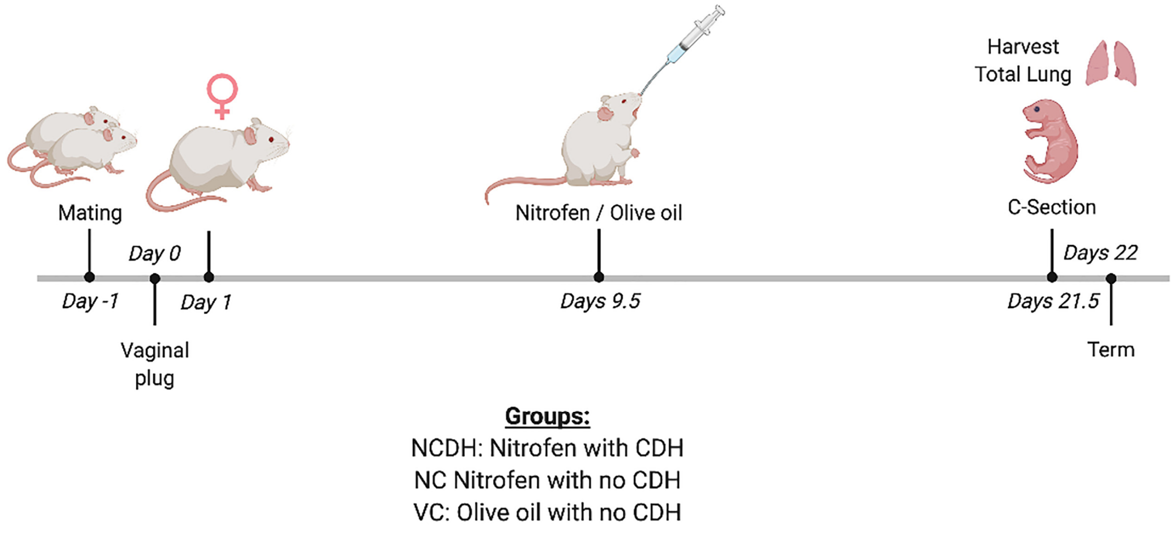

Figure S5. Nitrofen CDH rat model. Mating date was defined as E-1 and plug date as 0. On E9.5 first group of pregnant rats received intragastrically 100 mg of Nitrofen dissolved in 1 mL of olive and the second group only olive oil. The diaphragm was inspected for the presence of CDH on E21.5. The pups were classified in the first group as Nitrofen CDH (NCDH) if they have CDH or Nitrofen control (NC) if CDH wasn't present. The second group are the vehicle control (VC).
